# The Receptor Tyrosine Kinase FGFR2b/KGFR Controls Early Differentiation of Human Keratinocytes

**DOI:** 10.1371/journal.pone.0024194

**Published:** 2011-09-21

**Authors:** Francesca Belleudi, Valeria Purpura, Maria Rosaria Torrisi

**Affiliations:** 1 Dipartimento di Medicina Clinica e Molecolare, Istituto Pasteur-Fondazione Cenci Bolognetti, Sapienza Università di Roma, Rome, Italy; 2 Azienda Ospedaliera S. Andrea, Rome, Italy; Virginia Commonwealth University, United States of America

## Abstract

The FGFRs trigger divergent responses, such as proliferation and differentiation, and the cell type as well as the context-dependent signaling are crucial for the functional outcome. The FGFR2b/KGFR is expressed exclusively on epithelial cells and plays a key role in skin homeostasis. Here we analyzed in vitro the role of KGFR in the early differentiation of keratinocytes modulating its expression by KGFR cDNA transient transfection or KGFR siRNA microinjection and inducing a synchronous wave of differentiation in pre-confluent cells. Immunofluorescence, biochemical and molecular approaches demonstrated that KGFR overexpression increased the early differentiation marker keratin 1 at both transcriptional and translational levels, while receptor depletion reduced it. Ligand-dependent receptor activation and signaling were required for this differentiative effect. Overexpression of kinase negative KGFR mutant or Tyr769 KGFR signaling mutant, which is not able to recruit and activate PLC-γ, showed that the receptor kinase activity, but not its PLCγ-mediated signaling, is required for differentiation. Reduction of K1 expression, obtained by AKT inhibition, demonstrated that the PI3K/Akt signaling pathway is involved in the control of KGFR-mediated keratinocyte differentiation. This in vitro experimental model indicates that FGFR2b/KGFR expression represents a key event regulating keratinocyte early differentiation during the switch from undifferentiated to differentiating cells.

## Introduction

The fibroblast growth factors receptors (FGFRs) are receptor tyrosine kinases (RTKs) expressed on many different tissues and involved in the control of different cellular key processes such as cell growth, differentiation, migration and survival (for a recent review see [1]). Even if the main FGFR-mediated signaling substrates and pathways are quite similar, numerous studies have demonstrated that FGFR activation can trigger divergent responses such as proliferation and differentiation depending on the cell type as well as the cellular context [1].

The keratinocyte growth factor receptor (KGFR/FGFR2b) is a splicing transcript variant of the fibroblast growth factor receptor 2 (FGFR2) expressed exclusively on epithelial cells [2] and activated by the specific high affinity binding of keratinocyte growth factor (KGF/FGF7) and fibroblast growth factor-10 (FGF10) [3], [4]. Secreted by dermal fibroblasts, both ligands promote the early differentiation program in human keratinocytes [5], [6]. Some reports have suggested a key role for KGFR expression in the skin homeostasis [7]–[9], regulating the balance between proliferation and differentiation; in fact, mice lacking the KGFR in keratinized epithelia display altered cell proliferation in the basal layer and compromised late differentiation, although the expression of early differentiation markers, such as K1, does not seem to be profoundly affected [7]–[9]. However, the results obtained in these “in vivo” models appeared frequently discordant and not conclusive, at least concerning the proliferative ability of the keratinocytes when KGFR is knocked out. Consistent with this statement, Yang et al. [9] have very recently demonstrated that the hyperproliferative effect induced by the lack of FGFR1b and FGFR2b/KGFR observed “in vivo” in KO mice was not confirmed in the corresponding “in vitro” model of cultured keratinocytes derived from these mice: this finding has been explained by the fact that, in the “in vivo” models, many microenvironmental factors, such as the presence of inflammatory components, may act hiding the specific functions of the receptors in skin homeostasis. This appears to suggest that the role of FGFR2b/KGFR expression in the regulation of keratinocyte differentiation cannot be properly investigated “in vivo”. On the other hand, the use of “in vitro” models has been particularly appropriated for the demonstration of the key role of KGFR as a tumour suppressor controlling epithelial cell differentiation: in fact, several studies have demonstrated that the re-expression of KGFR in cultured cells from epithelial tumours in which this receptor is down-regulated was able to inhibit cell growth and to induce differentiation [10]–[14]. Thus, to evaluate the single contribution of KGFR expression in both the induction of keratinocyte differentiation and in the maintenance of this process in cells already committed to differentiate, we have believed useful to develop here an “in vitro” cellular model in which the modulation of the receptor expression, as well as the differentiation process, could be highly controlled and easily monitored. We have thought that a rapid and synchronous modulation of the receptor expression could be efficiently obtained in cultured keratinocytes by transient transfection of KGFR cDNA or by microinjection of KGFR siRNA, while a synchronous wave of differentiation in pre-confluent cells would be generated by treatment with Thapsigargin (TG), an inhibitor of endoplasmic reticulum Ca^2+^-ATPase pump family [15]. In addition, this strategy of KGFR modulation and forced cell differentiation would also permit to study the signaling pathways responsible for the differentiative response.

Among the possible candidates for the regulation of KGFR-mediated early differentiation in keratinocytes, the PI3K/Akt signaling pathway could be considered for several reasons: in fact, PI3K/Akt activity increases during keratinocyte differentiation [16], [17] and inhibition of the Akt pathway by RNA interference results in an altered epithelial stratification [17] In addition, it has been very recently demonstrated a possible role of the PI3K/Akt pathway in the KGFR-mediated differentiation process of pancreatic ductal to beta cells [18].

Another signaling pathway that might be involved in the KGFR-dependent differentiation is that mediated by PLC-γ: in fact, PLC-γ is able to hydrolyze PI(4,5)P2 to IP3 and DG second messengers triggering the calcium-induced differentiation response of human keratinocytes [19], [20]. Moreover, it has been recently demonstrated that the FGF2-dependent activation of PLC-γ is able to induce a sustained activation of MAPKs that in turn triggers transdifferentiation of bone marrow stromal cells in neuronal cells [21]. We and others have shown that the KGFR autophosphorylation site Y769 is required for PLC-γ binding and activation [22], [23]. Although the specific mutagenesis of this tyrosine site in phenylalanine (Y769F) has been used to analyze the role of PLC-γ in the KGFR-mediated proliferative signaling and response [22], [24], its effect on keratinocyte differentiation remains to be investigated.

To identify the possible receptor-mediated signaling pathways involved in the earlier induction of differentiation, we used here the KGFR signaling mutant Y769F (KGFR Y769F), that is not able to recruit and activate PLC-γ [22] and a kinase negative KGFR mutant (KGFR Y656F/Y657F) [25] as negative control. The possible involvement of the PI3K/Akt signaling pathway was also investigated through the use of a specific Akt inhibitor.

## Results

### The expression of KGFR regulates early differentiation

**Figure 1:**
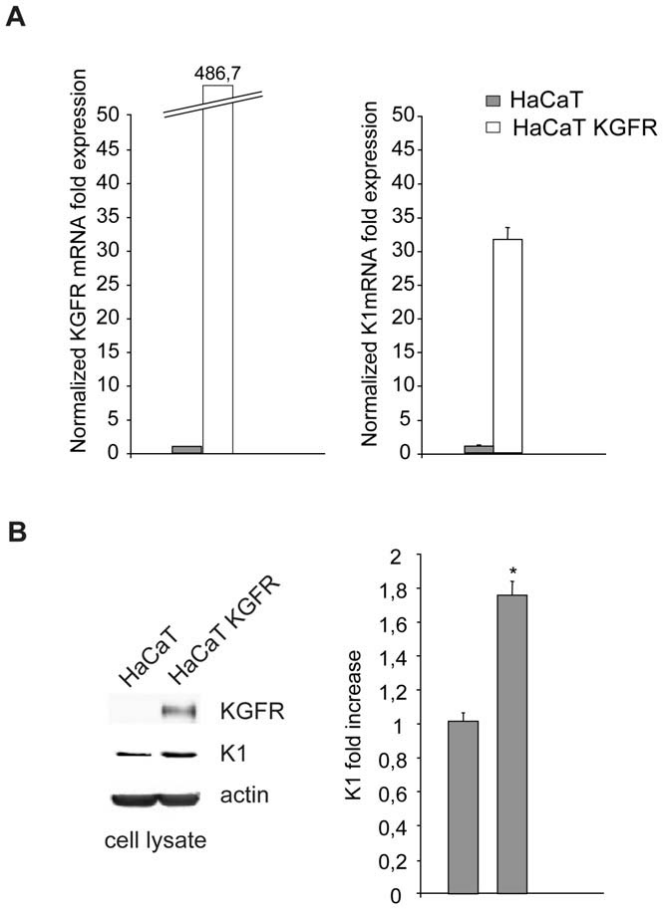
KGFR expression triggers the early differentiation in undifferentiated keratinocytes. (A) HaCaT cells were transfected with the pCI-neo expression vector containing human KGFR cDNA (HaCaT KGFR). After 24 h from transfection, the KGFR and K1 mRNA transcript levels were quantitated by real-time RT-PCR: a clear fold increase in both K1 mRNA (right panel) and KGFR mRNA (left panel) expression is observed in HaCaT KGFR cells compared to control ones. (B) Western blot analysis on HaCaT KGFR and HaCaT cells using anti-K1 monoclonal antibody and with anti-Bek polyclonal antibodies, which recognize the KGFR/FGFR2b protein, shows that the band of K1 is increased by KGFR overexpression in HaCaT KGFR compared to control cells. The specific band corresponding to KGFR protein is well visible only upon KGFR transfection. The equal loading was assessed with anti-actin antibody. For densitometric analysis of the band corresponding to K1 protein the values from three independent experiments were normalized, expressed as fold increase and reported in graph as mean values +/− standard deviation (SD). Student' t test was performed and significance level has been defined as *p<0.005 vs the corresponding untransfected cells.

The crucial role of keratinocyte growth factor receptor (KGFR/FGFR2b) and of its ligands in the control of the epithelial cell homeostasis has been widely demonstrated [26]. In addition, previous results from our group have demonstrated that KGFR is up-modulated during cell differentiation [27], [28], [29]. To analyze in detail the contribution of KGFR expression in the induction of keratinocyte early differentiation, we used the human keratinocyte HaCaT cell line, spontaneously immortalized from a primary culture of keratinocytes and widely used as a model of keratinocyte differentiation and stratification [30], [28]. Pre-confluent HaCaT cells expressing very low levels of KGFR were transiently transfected with KGFR (HaCaT KGFR) and we assessed the effect of its forced expression on the early differentiation marker keratin 1 (K1). Quantitative real-time RT-PCR showed a considerable increase in both K1 mRNA (Figure 1A, right panel) and KGFR mRNA (Figure 1A, left panel) expression at 24 hours from transfection in HaCaT KGFR cells compared to control cells. The enhanced expression of K1 induced by KGFR overexpression was also validated at the protein level by Western blot analysis using anti-K1 polyclonal antibodies, which showed that the band at the molecular weight corresponding to K1 protein was increased in HaCaT KGFR cells compared to control cells (Figure 1B). Immunoblot analysis using anti-Bek polyclonal antibodies, which recognize the intracellular portion of the two splicing variants KGFR/FGFR2b and FGFR2c, showed that the 140 KDa specific band corresponding to the receptor molecular weight was well visible only upon KGFR transfection (Figure 1B). The equal loading was assessed with anti-actin antibody and densitometric analysis was performed as described in materials and methods. These results indicated that, in pre-confluent HaCaT cells expressing very low levels of endogenous KGFR, the receptor overexpression up-regulates K1 at both transcriptional and translational levels, suggesting that increased expression of this receptor might be able to trigger early differentiation in undifferentiated keratinocytes.

**Figure 2:**
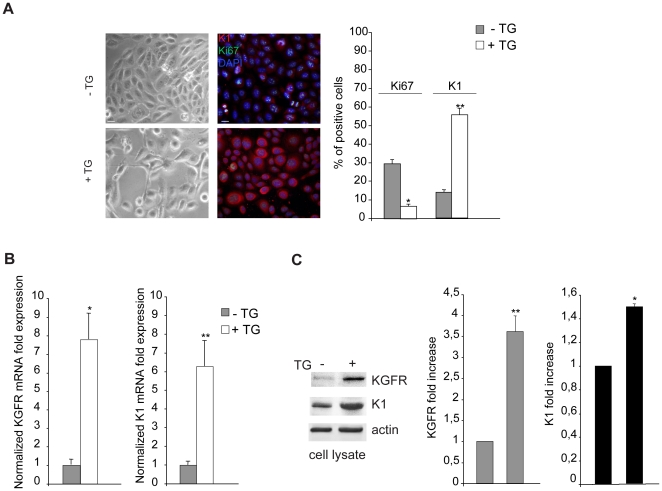
Thapsigargin-induced differentiation is associated with increased KGFR expression. (A) HaCaT cells were treated with TG 0.5 µM for 1 h at 37°C following by incubation at 37°C for 48 h. Cells treated with equal amount of DMSO solvent were used as a control. Phase contrast microscopy analysis shows that the TG-treated cells appear detached each other and elongated, while control cells are closely packed and polygonal (left panels). Quantitative immunofluorescence analysis using anti-Ki67 and anti-K1 antibodies shows that TG treatment significantly decreases the percentage of cells expressing the proliferative marker Ki67 and increases that of cells positive for the early differentiation marker (right panels). Cell nuclei were visualized by DAPI. The quantitative analysis was assessed by counting for each sample a total of 50 cells, randomly observed in 10 microscopic fields from three different experiments. Cut-off of the K1 signal intensity was determined for TG-treated and control samples as described in materials and methods. Results are expressed as mean values ± standard errors (SE). Student's t test was performed and significance level has been defined as *p<0.001 and **p<0.05 vs the corresponding untreated cells. (B) Quantitative real-time RT-PCR of KGFR and K1 mRNA transcript levels on TG-treated and control HaCaT cells shows an evident increase in both KGFR mRNA (left panel) and K1 mRNA (right panel) expression upon TG-treatment. Student's t test was performed and significance level has been defined as *p<0.005 and **p<0.001 vs the corresponding untreated cells. (C) Western blot analysis shows that the KGFR band, which is hardly detectable in control cells, becomes well visible in TG-treated cells. Enhancement of K1 protein expression is also evident upon TG treatment. The equal loading was assessed with anti-actin antibody. For densitometric analysis of the bands, the values from three independent experiments were normalized, expressed as fold increase and reported in graphs as mean values +/− SD. Student's t test was performed and significance level has been defined as *p<0.001 and **p<0.001 vs the corresponding untreated cells.

Then, we wondered if the KGFR overexpression could enhance early differentiation also in differentiating HaCaT cells expressing increased levels of endogenous KGFR. Since cell pre-confluence is required for transient transfection and, consequently, transient KGFR overexpression can not be maintained for the time necessary to permit HaCaT cell stratification [28], we induced the differentiation program in pre-confluent conditions using Thapsigargin (TG), an inhibitor of the endoplasmic reticulum Ca^2+^-ATPase that it is known to trigger HaCaT differentiation [15] inducing a dose-dependent release of stored Ca^2+^ in the cytoplasm [31], [32]. Pre-confluent HaCaT cells were treated with different doses of TG as reported in materials and methods and the number of living metabolically active cells was evaluated by MTT test measuring the absorbance at 570 nm by a spectrofotometer (data not shown). Based on this preliminary set-up, we found that, taking in account both cell differentiation and cell viability, the best condition was obtained at the dose of 0.5 µM TG followed by incubation at 37°C for 48 h (data not shown). Control cells were treated with equal amount of the solvent dimethyl sulfoxide (DMSO). The differentiative effect of the selected dose of TG was first analyzed by phase contrast microscopy: while cells treated with DMSO alone appeared non differentiated, closely packed and polygonal (Figure 2A, left panels), the TG-treated cells showed morphological changes characteristic of keratinocyte differentiation [33]: in fact, the cells appeared detached each other and more elongated if compared to control cells (Figure 2A, left panels). The proliferation rate and the early differentiation were assessed by immunofluorescence using anti-Ki67 monoclonal antibody and anti-K1 polyclonal antibodies respectively. Quantitative immunofluorescence analysis showed that, consistent with the differentiative stimulus, TG treatment significantly decreased the percentage of cells expressing the proliferative marker Ki67 and increased that of cells positive for K1 (Figure 2A, right panels), as expected [15], [34]. The enhanced expression of K1 induced by TG was also demonstrated at both transcriptional and translational levels by real-time RT-PCR (Figure 2B) and Western blot analysis (Figure 2C), confirming the ability of TG to trigger HaCaT cell differentiation. In addition, immunoblot using anti-Bek antibodies showed that the band corresponding to KGFR, hardly detectable in control cells, was evident upon TG treatment despite the pre-confluent condition (Figure 2C) and correlated with a clear increase of KGFR mRNA levels (Figure 2B). Thus, TG treatment allows to obtain a synchronous wave of differentiation and is able to generate a homogenous population of HaCaT differentiating cells expressing increasing amount of KGFR.

**Figure 3:**
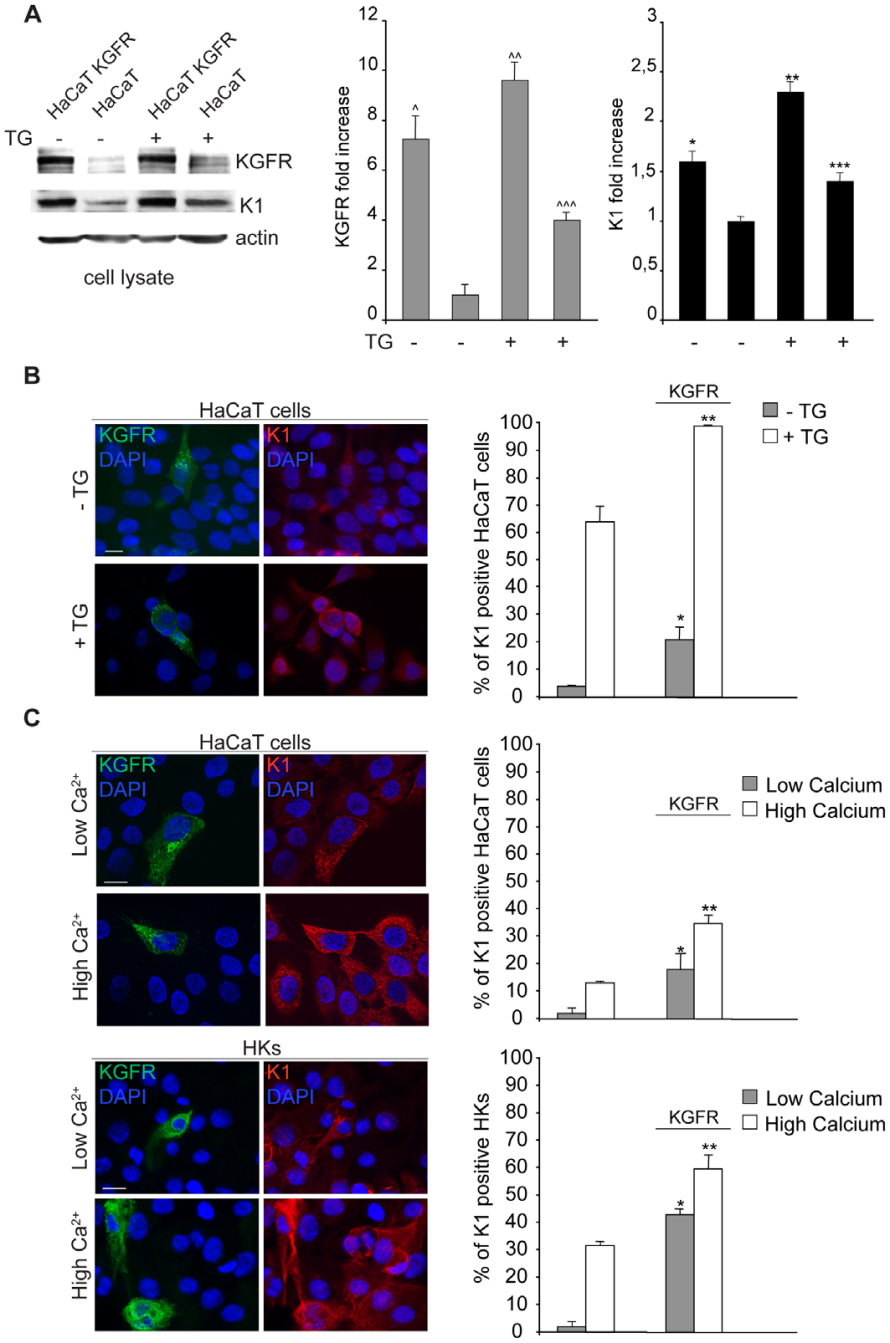
KGFR expression enhances early differentiation in differentiating keratinocytes. HaCaT KGFR transfected cells and HaCaT control cells were treated with TG as above, while the control cells were kept in DMSO alone. (A) Western blot followed by densitometric analysis, as described in Figure 2C, shows that the strong increase of the KGFR band in transfected cells corresponds to a parallel enhancement of the K1 protein levels in both TG-treated or untreated samples. Student's t test was performed and significance level has been defined as ∧p<0.001 Vs the corresponding untranfected cells, ∧∧p<0,05 and ∧∧∧p<0,005 vs the corresponding TG-untreated cells, *p<0,005 vs the corresponding untransfected cells, **p<0.05 and ***p<0.005 vs the corresponding TG-untreated cells (B) Double immunofluorescence staining with anti-Bek and anti-K1 antibodies shows that the signal of the transfected receptor appears localized either on the plasma membrane and in intracellular compartments. Quantitative analysis shows that KGFR overexpression significantly increases the percentage of cells expressing K1 in both TG-treated and control cells. (C) HaCaT cells and primary cultured HKs were transiently transfected with KGFR and induced to differentiate by incubation with high Ca^2+^ concentration (2 mM for HaCaT cells and 1.5 mM for HKs) in the medium or kept in low Ca^2+^ (0.1 mM for HaCaT cells and 0.03 mM for HKs). Double immunofluorescence analysis performed as above and its quantitation confirms the effect of KGFR overexpression in increasing the percentage of K1 positive cells in either high Ca^2+^ or low Ca^2+^ conditions. The quantitative analysis in B and C was assessed counting for each sample a total of 50 cells overexpressing KGFR randomly observed in 10 microscopic fields from three different experiments and comparing them with the surrounding cells that do not display receptor overexpression. Cut-off of K1 signal intensity was determined as above. Results are expressed as mean values ± SE. Student's t test was performed and significance level has been defined as *p<0.001 and **p<0.01 vs the corresponding surrounding cells that do not show KGFR overexpression (B), *p<0.05 and **p<0.05 (C: HaCaT cells) and *p<0.005 and **p<0.05 (C: HKs) vs the corresponding surrounding cells that do not show KGFR overexpression. Bars, 10 µm.

Therefore, having the appropriate tool to induce differentiation in our model system, in order to assess if KGFR overexpression could enhance early differentiation in HaCaT cells undergoing differentiation, we analyzed the effect of KGFR transfection on K1 expression in cells forced to synchronously differentiate by TG treatment. To this aim, pre-confluent HaCaT cells were transiently transfected with KGFR and then treated with TG, while the control cells were kept in DMSO alone: samples were then processed for biochemical or immunofluorescence analysis. Western blot performed as above revealed that the increased expression of KGFR in transfected cells corresponded to a parallel enhancement of the K1 protein levels also upon TG treatment (Fig. 3A), indicating that the effect mediated by receptor expression is well detectable in cells induced to differentiate. Double immunofluorescence using anti-Bek monoclonal antibody and anti-K1 polyclonal antibodies showed that the signal of the transfected receptor appeared localized both on the cell surface and in intracellular compartments, as expected [25]. The quantitative analysis showed that the overexpression of KGFR was able to significantly increase the percentage of cells expressing K1 in both TG-treated and control cells (Figure 3B). Thus, KGFR plays a crucial role in the control of early differentiation, since its expression triggers early differentiation in undifferentiated cells as well as enhances this process in cells already committed to differentiation. To confirm the physiological role of KGFR in this process in a well established cell model of keratinocyte differentiation, we repeated the experiments described above using high extracellular calcium addition as an alternative stimulator of differentiation in both HaCaT cells and primary cultures of normal human keratinocytes (HKs). Cells were transiently transfected with KGFR and induced to differentiate in response to increased Ca^2+^ concentration (2 mM for HaCaT cells [35], 1.5 mM for HKs) in the medium or kept in low Ca^2+^ medium (0.1 mM for HaCaT cells, 0.03 mM for HKs). Quantitative immunofluorescence analysis as above showed that, also upon extracellular Ca^2+^ treatment and in primary keratinocytes, the KGFR expression induces an increase of K1 positive cells (Fig. 3C), indicating that the KGFR role in the regulation of the early differentiation represents a physiological general phenomenon in epidermal cells.

**Figure 4:**
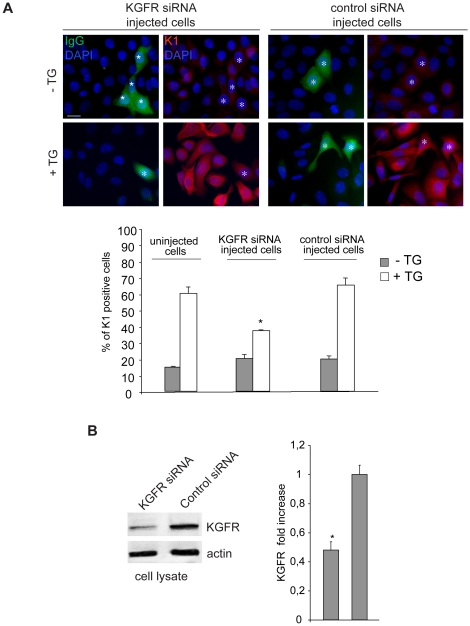
KGFR/FGFR2b depletion inhibits early differentiation in keratinocytes. (A) HaCaT cells were coinjected with KGFR siRNA to obtain KGFR silencing and mouse IgG to identify the microinjected cells. Control cells were injected with an unrelated siRNA. After injection, cells were treated with TG as above. Quantitative immunofluorescence analysis using anti-K1 polyclonal antibodies shows that the percentage of K1 positive cells in KGFR-depleted and TG-treated cells is significantly decreased if compared either to uninjected cells in the same microscopic field or to cells injected with control siRNA. The quantitative analysis was assessed as described in figure 2D. Results are expressed as mean values ± SE. Student's t test was performed and significance level has been defined as *p<0.001 vs the corresponding uninjected cells. Bar, 10 µm. (B) Western blot analysis with anti-Bek polyclonal antibodies in HaCaT cells transfected with KGFR siRNA or with the control unrelated siRNA and treated with TG as above. The KGFR protein expression is down-regulated in KGFR siRNA-transfected cells. The equal loading was assessed with anti-actin antibody. For densitometric analysis of the band corresponding to KGFR protein, the values from three independent experiments were normalized, expressed as fold increase and reported in graph as mean values +/− SE. Student's t test was performed and significance level has been defined as *p<0.01 vs the corresponding uninjected cells.

To unequivocally demonstrate the specific direct function of KGFR in regulating the early differentiation, we analyzed the effect of the receptor depletion on K1 expression. To this aim, we performed co-injection of small interfering RNA for FGFR2/Bek (KGFR siRNA) to obtain receptor silencing and mouse IgG to identify the microinjected cells. Microinjection with an unrelated siRNA was performed as a control. After injection, cells were treated with TG or kept in DMSO and then warmed to 37°C as above. Quantitative immunofluorescence analysis showed that in TG-treated cells expressing increased levels of KGFR protein, as stated above (Figure 2C), the KGFR siRNA injection and consequent receptor depletion induced a significant decrease in the percentage of K1 positive cells compared to the uninjected ones surrounding them in the same microscopic fields or to cells injected with control siRNA (Figure 4A). In contrast, in cells kept with DMSO alone expressing very low levels of endogenous KGFR protein (Figure 2C), the low percentage of K1 positive cells appeared unaffected by KGFR siRNA injection (Figure 4A). The efficiency of KGFR depletion was evaluated by transfecting HaCaT cells with KGFR siRNA or with a control siRNA and then treating them with TG as above. Western blot analysis using anti-Bek polyclonal antibodies showed that KGFR protein expression appeared down-regulated in KGFR siRNA-transfected cells (Figure 4B). The equal loading was assessed with anti-actin antibody. Taken together, these results demonstrate that the modulation of the receptor expression is crucial in regulating cell differentiation.

### KGFR signaling through PI3K/Akt pathway, but not through PLC-γ activation, is involved in the induction of early differentiation

**Figure 5:**
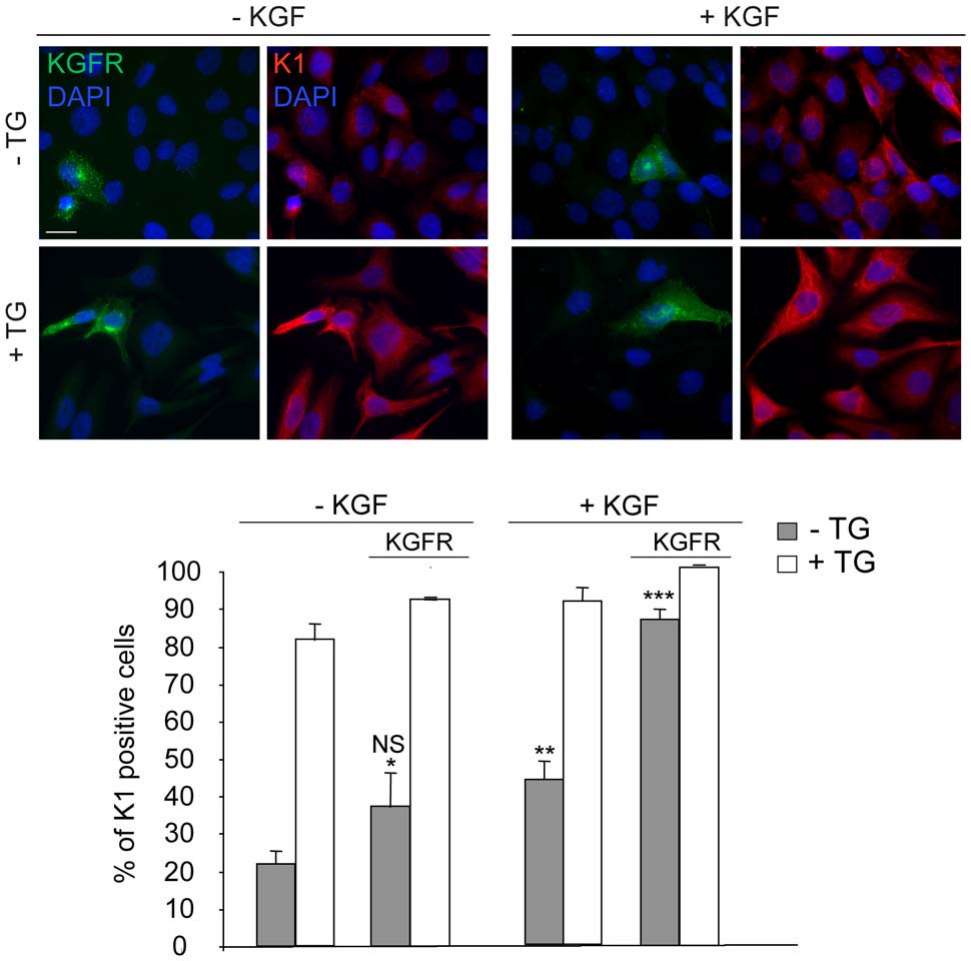
KGF-induced KGFR activation is required for the induction of early differentiation. HaCaT KGFR cells were treated with TG as above, serum starved for 12 h and stimulated the last 24 h at 37°C with 20 ng/ml KGF. Quantitative immunofluorescence analysis performed as described in figure 2D shows that KGF stimulation increases the percentage of K1 positive cells. This KGF-induced increase is higher in cells overexpressing KGFR compared to cells expressing endogenous receptor levels and is particularly evident in undifferentiated cells, while the TG-treated cells are almost all K1 positive independently on KGF addition. No significant increase in the percentage of K1 positive cells is induced by KGFR overexpression in KGF untreated cells as a consequence of serum deprivation. Results are expressed as mean values ± SE. Student's t test was performed and significance level has been defined as *: NS vs the corresponding surrounding cells that do not display KGFR overexpression, **p<0.01 and ***p<0.001 vs the corresponding unstimulated cells. Bar, 10 µm.

To first analyze if the ligand-dependent activation of KGFR is required for the induction of early differentiation, HaCaT KGFR cells treated with TG or kept in DMSO alone as above were serum starved and stimulated the last 24 h at 37°C with KGF 20 ng/ml. Quantitative immunofluorescence analysis using anti-Bek and anti-K1 antibodies showed that KGF stimulation induced an increase in the percentage of K1 positive cells as expected [27], [28] and this KGF-induced increase was higher in cells overexpressing KGFR compared to cells expressing endogenous receptor levels (Figure 5). This enhancement was particularly evident in undifferentiated cells, because the TG-treated cells were almost all K1 positive independently on KGF addition. Interestingly, in KGF untreated cells, no significant increase in the percentage of K1 positive cells was induced by KGFR overexpression (see Figure 5 compared to Figure 3B) as a consequence of serum deprivation performed in these set of experiments, further confirming the requirement of KGFR activation and signaling in this process.

**Figure 6:**
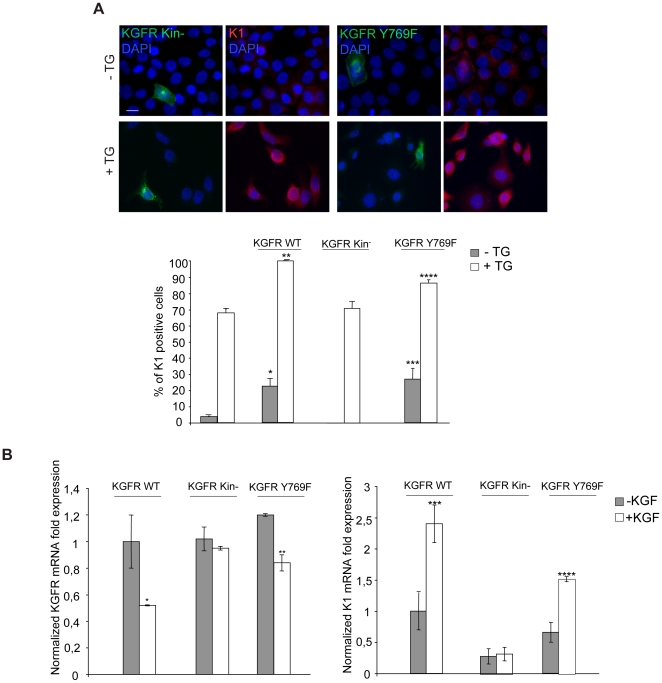
KGFR-mediated early differentiation is independent on PLC-γ recruitment and activation. (A) HaCaT cells transiently transfected with KGFR WT, with KGFR Y769F signaling mutant or with KGFR Y656F/Y657F kinase dead mutant were treated with TG as above. Quantitative immunofluorescence analysis performed as described in figure 2D shows that, similarly to KGFR WT, KGFR Y769F overexpression significantly increases the percentage of cells expressing K1 either in TG-treated and in the control cells, while the KGFR Y656F/Y657F overexpression does not affect it. Results are expressed as mean values ± SE. Student's t test was performed and significance level has been defined as *p<0.001, **p<0.01, ***p<0.001 and ****p<0.05 vs the corresponding surrounding cells that do not display KGFR overexpression. Bar, 10 µm. (B) HaCaT cells were transfected and treated with TG and KGF as above. KGFR mRNA (left panel) and K1 mRNA (right panel) transcript levels were quantitated by real-time RT-PCR: a ligand-dependent increase in K1 mRNA expression is observed in cells transfected with KGFR WT and with KGFR Y769F but not in cells transfected with KGFR Y656F/Y657F. A ligand-dependent down-modulation in KGFR mRNA expression is observed in HaCaT KGFR WT cells and in HaCaT KGFR Y769F cells but not in KGFR Y656F/Y657F cells. Student's t test was performed and significance level has been defined as *p<0.001, **p<0.05, ***p<0.005 and ****p<0.001 vs the corresponding unstimulated cells.

Since it has been demonstrated that PLC-γ activation controls keratinocyte differentiation through PI(4,5)P2 hydrolysis and generation of the second messengers IP3 and DG [19], [20], we wondered if KGFR-mediated activation of PLC-γ might be involved in the induction of HaCaT cell early differentiation. To address this point, cells were transiently transfected alternatively with KGFR WT or with a KGFR signaling mutant in which the tyrosine 769, required for PLC-γ binding and activation [22], [23] has been substituted by phenylalanine (Y769F) [22]. In addition, cells were transiently transfected with a Y656F/Y657F KGFR kinase dead mutant [25] as a control. After transfection, cells were treated with TG or kept in DMSO alone as above. Quantitative immunofluorescence analysis using anti-Bek and anti-K1 antibodies showed that, similarly to KGFR WT, KGFR Y769F overexpression was able to significantly increase the percentage of cells expressing K1 either upon treatment with TG or in the presence of DMSO alone (Figure 6A). In contrast, the overexpression of the kinase negative mutant KGFR Y656F/Y657F did not affect the early differentiation, confirming the role of the receptor signaling (Figure 6A). Quantitative real-time RT-PCR performed in HaCaT cells transiently transfected with KGFR WT or KGFR mutants, and then treated with TG and KGF as above, showed a significant ligand-dependent increase in K1 mRNA expression in HaCaT KGFR WT and HaCaT KGFR Y769F, but not in HaCaT KGFR Y656F/Y657F cells (Figure 6B, right panel). Moreover, we observed a ligand-dependent decrease in KGFR mRNA expression in both HaCaT KGFR WT and HaCaT KGFR Y769F, but not in HaCaT KGFR Y656F/Y657F, due to the receptor internalization and consequent down-modulation induced by KGF (Figure 6B, left panel). These results indicate that, although the KGFR kinase activity is required for the keratinocyte differentiation, recruitment and consequent activation of the receptor substrate PLC-γ do not appear to play a role in this process.

**Figure 7:**
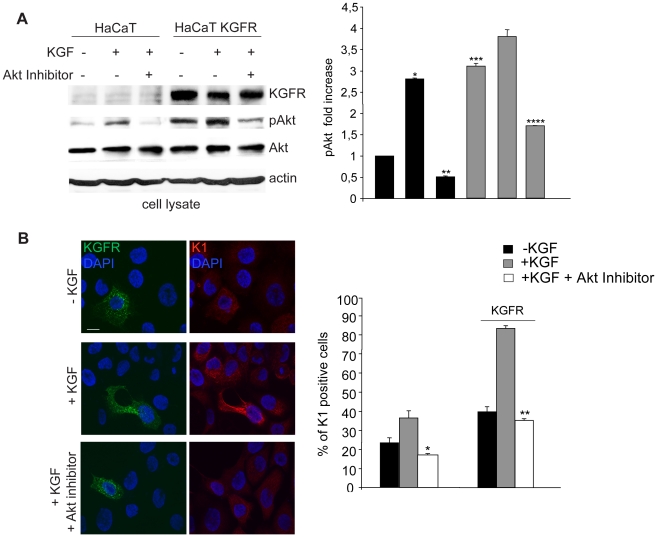
The PI3K/Akt signaling pathway is involved in KGFR-mediated early differentiation. (A) HaCaT KGFR and HaCaT cells were serum starved for 4 h and treated with 100 ng/ml KGF for 10′ at 37°C. Western blot analysis using anti-Bek and anti-phospho-Akt polyclonal antibodies shows that Akt protein phosphorylation is evident following KGF treatment and it is specifically blocked by the Akt inhibitor in HaCaT control cells. In KGFR transfected cells, the Akt phosphorylation is more intense and clearly detectable also in serum-free untreated cells. The specific band corresponding to the receptor is well visible only in KGFR transfected cells. The equal loading was assessed with anti-actin antibody. For the densitometric analysis of band corresponding to phospho-Akt protein band the values from three independent experiments were normalized, expressed as fold increase and reported in graph as mean values +/− SE. Student's t test was performed and significance level has been defined as *p<0.001 vs the corresponding KGF-untreated cells, **p<0.005 vs the corresponding Akt inhibitor-untreated cells, ***p<0.001 vs the corresponding untransfected cells and ****p<0.005 vs the corresponding Akt inhibitor-untreated cells. (B) HaCaT KGFR and HaCaT cells were serum starved for 12 h and then stimulated with 20 ng/ml KGF for the last 24 h at 37°C in presence or not of the specific Akt inhibitor. Quantitative immunofluorescence analysis shows that the Akt inhibitor drastically blocks the increase of K1 positive cells observed upon KGFR overexpression and KGF stimulation. The Akt inhibitor decreases the KGF-mediated differentiation also in cells expressing endogenous levels of the receptor. Results are expressed as mean values ± SE. Student's t test was performed and significance level has been defined as *p<0.001 and **p<0.001 vs the corresponding, KGF-treated cells. Bar, 10 µm.

It has been previously reported that the PI3K/Akt signaling is important in the regulation of keratinocyte differentiation [16] and it might provide an essential survival signal required for keratinocyte stratification and differentiation [17]. In addition, it has been very recently demonstrated a possible role of the PI3K/Akt pathway in the KGFR-mediated differentiation process of pancreatic ductal cells to beta cells [18]. To clarify if the PI3K/Akt could be the main differentiative pathway induced by KGFR expression and signaling, first HaCaT cells were transfected with KGFR and then stimulated with KGF (100 ng/ml for 10′ at 37°C) to induce receptor activation as previously reported [25], [36] and the amount of Akt phosphorylation was assessed by Western blot analysis using anti-pAkt antibody. As shown in Figure 7A, in HaCaT control cells, Akt protein phosphorylation was evident following KGF treatment and was specifically blocked by the Akt inhibitor. In KGFR transfected cells, the Akt phosphorylation appeared more intense and clearly detectable also in serum-free untreated cells, possibly as a result of the short time starvation performed in these experiments. In fact, trans-phosphorylation of overexpressed KGFRs can be excluded, based on previous reported evidences [25], [37].

To demonstrate if the PI3K/Akt pathway activated by KGFR could be responsible for the induction of the early differentiative program, we serum starved HaCaT KGFR cells and treated them for the last 24 h at 37°C with KGF as above to induce K1 expression in presence or not of the specific Akt inhibitor. Quantitative immunofluorescence analysis showed that the Akt inhibitor drastically blocked the increase of K1 positive cells observed upon KGFR overexpression and KGF stimulation (Fig. 7B). Moreover, the Akt inhibitor was able to significantly decrease the KGF-mediated differentiation also in cells expressing endogenous levels of the receptor (Figure 6B). Thus, these results indicate that the early differentiation induced by KGFR expression occurs through the Akt signalling pathway.

## Discussion

The possible key role of FGFR2b/KGFR in controlling cell differentiation stems mostly on the fact that this receptor is down-modulated in several epithelial tumors (for a recent review see [26]) and that its re-expression alone is able to induce cell differentiation [10], [13], [14]. However, only a few reports have suggested that the expression of the receptor might be crucial in the normal cell physiology of the epithelial differentiation [7]–[9] although this possibility is sustained by evidences that the expression of other FGFRs, such as FGFR1 and FGFR3 [21], [38], [39] or other receptor tyrosine kinases, such as TrkA [40], can be directly involved in the regulation of cell growth arrest and differentiation. With the aim to elucidate the role of KGFR in the differentiative process, we used a controlled system of human keratinocytes led to express K1 and to change their morphology in a synchronized manner through the treatment with thapsigargin. In addition, in our model system the treatment with thapsigargin was also able to generate a homogeneous population of differentiating cells expressing increasing amounts of KGFR, allowing us to analyze the effects of a synchronized enhancement in the expression levels of endogenous receptor.

Among the well known different stages which characterized the sequential differentiation of the epidermal keratinocytes, we focused our attention on the earlier differentiation step, because it has been previously demonstrated by our group and by others that KGFR and K1 expression are increased during the transition from basal to suprabasal cells [27], [28], [41] and that this step is critical for the response to the ligand KGF [42]. We modulated here KGFR expression by cDNA transient transfection or by siRNA microinjection in the HaCaT human keratinocyte cell line, which are immortalized and warrant a constant and reproducible behaviour in vitro, and we evaluated its effects on early differentiation monitoring K1 expression by immunofluorescence and molecular approaches. In addition, through the rapid increase of intracellular Ca^2+^ released in response to thapsigargin, as well as through the increase in extracellular Ca^2+^ by its addition to the medium, we could compare on pre-confluent cells the effects of KGFR modulation in undifferentiated versus differentiating keratinocytes. Our results demonstrated that the forced expression of KGFR alone is able to increase K1 expression at both transcriptional and translational levels, which suggests that the receptor triggers and controls early differentiation in undifferentiated as well as in differentiating keratinocytes, in agreement with the previous reports on transformed epithelial cells [10], [13], [14]. Moreover, differently from what reported by Petiot et al. [7] Grose et al. [8] and Yang et al. [9] in KGFR KO mouse models, we found that the receptor depletion by KGFR siRNA microinjection reduced K1 expression. This contrasting result may be explained considering that the “in vivo” and “in vitro” models represent quite different contexts. In fact, growth factors and cytokines, such as those released by an inflammatory or wound healing microenvironment, may act simultaneously and may interfere with the activity of KGFR, possibly hiding its single contribution. In agreement with our hypothesis, Yang et al. [9] have very recently demonstrated that the hyperproliferative effect of FGFR1b and FGFR2b/KGFR depletion observed in the basal layer of the KO mice skin was not found in cultured keratinocytes isolated from these mice tissues, since hyperproliferation could be an indirect effect of the inflammation derived from the reduced epidermal barrier function. Thus, we believed that our “in vitro” experimental model is suitable to demonstrate that the modulation of KGFR expression is a specific key event regulating K1 expression and consequently the keratinocyte early differentiation during the switching from undifferentiated to differentiating cells.

To further elucidate the receptor role, we wondered whether the differentiative role of KGFR implies receptor activation and signaling. Since it has been shown that FGFR2b/KGFR overexpression in epithelial cells does not induce receptor trans-phosphorylation [25], [37], the results obtained here by serum-starving HaCaT KGFR cells and then treating them with KGF have unequivocally demonstrated that ligand-dependent KGFR activation is required for the receptor function in the induction of early differentiation. These results are also in agreement with those obtained by Zhang et al. [13] and Feng et al. [10] in nude mice tumors derived from implants of epithelial tumor cells, in which the down modulated KGFR was exogenously re-expressed leading to slow down the tumor growth and to enhance cellular differentiation: in fact, the importance of stroma cells, producing KGF, in the induction of epithelial tumor cell differentiation was clearly underlined in these papers. Moreover, comparing the effects of the overexpression of the receptor kinase dead mutant KGFR Y656F/Y657F with those obtained overexpressing KGFR WT, we further demonstrated that the intrinsic receptor tyrosine kinase activity is required for the differentiative function.

Since our present results pointed out the importance of the ligand-dependent KGFR signaling in the control of keratinocyte differentiation, we then focused our attention on the possible signaling pathway that might be involved in the induction of KGFR-mediated differentiation. Current knowledge points to the MAPK pathway as the downstream FGFR signaling involved in the differentiative outcome, suggesting that differentiation might be induced by a FGFR-mediated sustained MAPK signaling [1], [43], [44]. Because it has been also proposed that the PLC-γ signaling pathway is required for the FGFR-mediated sustained activation of the MAPKs [21] and that this substrate is able to trigger the calcium-induced differentiation response in human keratinocytes [1], [20], in this paper we investigated if PLC-γ signaling could be the main KGFR-mediated differentiative pathway in keratinocytes. To this aim, we overexpressed a KGFR Y769F signaling mutant, which is not able to recruit and activate PLC-γ [22] and we found that this mutant receptor acts similarly to the WT receptor in triggering the expression of K1, implying that this signaling pathway is not involved in the KGFR-induced early differentiation.

Because it has been reported that the Akt phosphorylation plays an important role in keratinocyte differentiation [16] and that the PI3K/Akt signaling might induce a survival outcome crucial for keratinocyte stratification and differentiation [17], it was reasonable to investigate if the KGFR-mediated activation of PI3K/Akt pathway could be required for the induction of keratinocyte differentiation. The results obtained in the present paper showed that the KGF-dependent Akt phosphorylation/activation, which is inhibited by the specific Akt inhibitor, was more intense in cells overexpressing KGFR; moreover, the Akt inhibitor significantly reduced the early differentiation induced by KGF in both non transfected HaCaT cells and in HaCaT KGFR transfected cells, suggesting that this pathway is involved in the KGFR-mediated keratinocyte differentiation. These results are in agreement with those obtained by Uzan et al. [18] showing that the KGF-mediated differentiation of pancreatic cells is controlled by the PI3K/Akt signaling pathway. Similarly, the possible involvement of Akt signaling in the cell growth arrest and differentiation induced by other members of the FGFR family has been proposed [45], even if the topic is still debated [40], [46].

In FGFRs, the PI3K/Akt signaling starts from the phosphorylation of the docking adaptor platform FRS2α (for a recent review, see [44]), in which six tyrosine residues are critical in determining the cellular context-specific signaling outcome. In a previous study from our group, we have observed that, during keratinocyte differentiation, the expression of FRS2α and Akt, as well as the Akt phosphorylation, was increased parallel to that of KGFR and K1, [47], already suggesting that this pathway would be more active in differentiated cells. Thus, we can conclude that KGFR plays a key role in the regulation of early stages of the keratinocyte differentiation program and that the up-modulation of this receptor in differentiating suprabasal keratinocytes induces the K1 expression probably through a profile-specific phosphorylation of FRS2α.

## Materials and Methods

### Cells and treatments

The human keratinocyte cell line HaCaT [30] was cultured in Dulbecco's DMEM, supplemented with 10% fetal bovine serum (FBS) plus antibiotics. HaCaT cells were transiently transfected with the pCI-neo expression vector containing human KGFR WT (HaCaT KGFR WT) or a kinase negative mutant KGFR Y656F/Y657F (HaCaT KGFR Kin^−^) or a signaling mutant KGFR Y769F (HaCaT KGFR Y769F) [25] using jetPEI™ DNA Trasfection Reagent (Polyplus-trasfection, New York, NY, USA) according to manufacturer's instructions.

For RNA interference and KGFR silencing, HaCaT cells were transfected with Bek small interfering RNA (Bek siRNA) (Santa Cruz Biotechnology Inc., Santa Cruz, CA, USA), or with unrelated siRNA as a control, using Lipofectamine 2000 Transfection Reagent (Invitrogen, Carlsbad, CA, USA) according to the manufacturer's protocol.

For growth factor stimulation, cells were serum starved for 12 h and then incubated with 20 ng/ml KGF (Upstate Biotechnology, Lake Placid, NY, USA) for 24 h at 37°C. Alternatively, to induce KGFR activation and signaling, cells were serum starved for 4 h and incubated with 100 ng/ml KGF (Upstate) for 10 minutes at 37°C. To inhibit Akt, cells were incubated with the specific Akt inhibitor 1L-6-hydroxy-methyl-chiro-inositol 2-(R)-2-O-methyl-3-O-octadecylcarbonate (Calbiochem, San Diego, CA) 1 µM for 1 h at 37°C before treatment with KGF in the presence of the inhibitor. To induce the differentiation program in pre-confluent conditions, HaCaT cells were incubated with different doses of Thapsigargin (TG) (0,1 µM, 0.5 µM, 1 µM and 2,5 µM) for 1 h at 37°C and followed by incubation at 37°C for 48 h. Since TG stock (1 mg/ml) was diluted in solvent dimethyl sulfoxide (DMSO), control cells were treated with an equal amount of DMSO. Alternatively, to induce differentiation with high extracellular calcium [35], cells were placed in medium containing 2 mM Ca^2+^.

Primary cultures of normal human keratinocytes (NHKs) were derived from skin biopsies and maintained in Medium 154-CF (Cascade Biologics, Portland, OR, USA) supplemented with Human Keratinocyte Growth Supplement (HKGS, Cascade Biologics) plus antibiotics and Ca^2+^ 0,03 mM (CascadeBiologics Inc.). To induce cell differentiation, primary human keratinocytes were placed in medium containing Ca^2+^ 1,5 mM.

### Microinjection

Microinjection was performed with an Eppendorf microinjector (Eppendorf, Hamburg, Germany) and an inverted microscope (Zeiss, Oberkochen, Germany). Injection pressure was set at 80–100 hPa and the injection time at 0.5 s. For KGFR depletion, a mixture of 100 nM KGFR small interfering RNA (Bek siRNA) (Santa Cruz Biotechnology) and 1 mg/ml mouse IgG (Cappel Research Products, Durham, NC, USA) in distilled water was microinjected in the cytoplasm of HaCaT cells to induce RNA interference and consequent KGFR silencing. Unrelated siRNA was microinjected as negative control. Cells were then treated with TG as above and processed for immunofluorescence.

### Immunofluorescence

Cells, grown on coverslips and incubated or not with KGF as above, were fixed with 4% paraformaldehyde in PBS for 30 minutes at 25°C followed by treatment with 0.1 M glycine for 20 minutes at 25°C and with 0.1% Triton X-100 for additional 5 minutes at 25°C to allow permeabilization. Cells were then incubated for 1 h at 25°C with the following primary antibodies: mouse monoclonal anti-Bek (1∶20 in PBS; C-8, Santa Cruz Biotechnology) which recognizes the KGFR/FGFR2b and FGFR2c isoforms, rabbit polyclonal anti-K1 (1∶50 in PBS; Covance, Emeryville, CA, USA) and mouse monoclonal anti-Ki67 (1∶50 in PBS; Novocastra, Newcastle-upon-Tyne, U.K.). The primary antibodies were visualized, after appropriate washing with PBS, using goat anti-mouse IgG-FITC (1∶20 in PBS; Cappel) and goat anti-rabbit IgG-Texas Red (1∶200 in PBS; Jackson Immunoresearch Laboratories, West Grove, PA, USA) for 30 minutes at 25°C. Nuclei were stained with DAPI (1∶1000 in PBS; Sigma). Coverslips were finally mounted with mowiol for observation. Fluorescence signals were analyzed by recording and merging stained images using a CCD device SPOT-2 camera (Diagnostic Instruments Inc., Sterling Heights, MI) and IAS2000/H1 software (Delta Sistemi, Roma, Italy). The fluorescence intensity of the signals was performed by the analysis of 50 cells for each sample in five different microscopic fields from three different experiments and the cut-off of the signal intensity was selected for both TG-treated and control samples in order to discriminate between K1 positive and negative cells using the KS300 3.0 Image Processing System (Zeiss, Oberkochen, Germany). Quantitative analysis of the percentage of Ki67 and/or K1-positive cells was assessed counting for each sample a total of 50 cells, randomly observed in 10 microscopic fields from three different experiments, except for the single experiment on HaCaT cells treated at low and high Ca^2+^ contrations. Results have been expressed as mean values ± standard errors (SE). p values were calculated using Student's t test and significance level has been defined as p<0.05.

### Western blot analysis

HaCaT and HaCaT KGFR cells were lysed in a buffer containing 50 mM HEPES pH 7.5, 150 mM NaCl, 1% glycerol, 1% Triton X-100, 1.5 mM MgCl_2_, 5 mM EGTA, supplemented with protease inhibitors (10 µg/ml aprotinin, 1 mM PMSF, 10 µg/ml leupeptin), and phosphatase inhibitors (1 mM sodium orthovanadate, 20 mM sodium pyrophosphate, 0.5 M NaF); 50 µg of total protein were resolved under reducing conditions by 8% SDS-PAGE and transferred to reinforced nitrocellulose (BA-S 83, Schleider and Schuell, Keene, NH, USA). The membranes were blocked with 5% non fat dry milk in PBS 0.1% Tween 20, and incubated with anti-Bek (C-17, Santa Cruz) polyclonal antibodies, anti-K1 (Covance) polyclonal antibodies, anti-phospho-Akt (Ser 473, Cell Signaling Technology, Beverly, MA) polyclonal antibodies (followed by enhanced chemiluminescence detection (ECL; Amersham, Alington Heights, IL). The membranes were rehydrated by being washed in PBS-Tween 20, stripped with 100 mM mercaptoethanol and 2% SDS for 30 min at 55°C, and probed again with anti-Akt 1/2 (H-136, Santa Cruz Biotechnology) polyclonal antibodies or anti-actin (Sigma) monoclonal antibody, to estimate the protein equal loading. Densitometric analysis was performed using Quantity One Program (Bio-Rad Laboratoires, Hercules, CA). Briefly, the signal intensity for each band was calculated and the background subtracted from experimental values. The resulting values from three different experiments were then normalized and expressed as fold increase respect to the control value.

### Primers

Oligonucleotide primers for target genes and for the housekeeping gene were chosen with the assistance of the Oligo 5.0 computer program (National Biosciences, Plymouth, MN) and purchased from Invitrogen. The following primers were used: for *FGFR2b/KGFR* target gene: 5′-CAGGGGTCTCCGAGTATGAA-3 (sense), 5′-TCTAAAGGCAACCTCCGAGA-3′ (anti-sense); for *K1* target gene 5′-AGCACAAGCCACACCACCATC-3′ (sense), 5′-CGCCACCTCCAGAACCATAGC-3′ (antisense); for the GAPDH housekeeping gene: 5′-CATCAGCAATGCCTCCTGCAC-3′ (sense), 5′-GTCATGAGTCCTTCCACGATACCAA-3′ (antisense).

For each primer pair, we performed no-template control and no-reverse-transcriptase control (RT negative) assays, which produced negligible signals.

### RNA extraction and cDNA synthesis

RNA was extracted using the TRIzol method (Invitrogen, Carlsbad, CA) according to manufacturer's instructions and eluted with 0,1% diethylpyrocarbonate (DEPC)-treated water. Total RNA concentration was quantitated by spectrophotometry and the quality was assessed by measuring the optical density ratio at 260/280 nm. RNA samples were stored at −80°C. After denaturation in DEPC-treated water at 70°C for 10 minutes, 1 µg of total RNA was used to reverse transcription using iScript™ cDNA synthesis kit (Bio-Rad) according to manufacturer's instructions.

### PCR amplification and real-time quantitation

Real-time PCR was performed using the iCycler Real-Time Detection System (iQ5 Bio-Rad) with optimized PCR conditions. The reaction was carried out in 96-well plate using iQ SYBR Green Supermix (Bio-Rad) adding forward and reverse primers for each gene and 1 µl of diluted template cDNA to a final reaction volume of 15 µl. All assays included a negative control and were replicated three times. The thermal cycling programme was performed as follows: an initial denaturation step at 95°C for 3 minutes, followed by 45 cycles at 95°C for 10 seconds and 60°C for 30 seconds. Real-time quantitation was performed with the help of the iCycler IQ optical system software version 3.0a (BioRad), according to the manufacturer's manual. The relative expression of the housekeeping gene was used for standardizing the reaction. The comparative threshold cycle (C_t_) method was applied to calculate the fold changes of expression compared to control cells Results are reported as mean ± standard deviation (SD) from three different experiments in triplicate.
